# Translating academic research into guidance to support healthcare improvement: how should guidance development be reported?

**DOI:** 10.1186/s12913-019-4792-8

**Published:** 2019-12-27

**Authors:** Simon Turner, Charlotte A. Sharp, Jessica Sheringham, Shaun Leamon, Naomi J. Fulop

**Affiliations:** 1 School of Management, University of Los Andes, Edificio Santo Domingo, Cl. 21 #1 20, Bogotá, Colombia; 2 Centre for Primary Care and Health Services Research, University of Manchester, Oxford Road, Manchester, M13 9PL UK; 3 Department of Applied Health Research, University College London, Gower Street, London, WC1E 6BT UK; 4 The Health Foundation, 8 Salisbury Square, London, EC4Y 8AP UK

**Keywords:** Guidance, Research evidence, Improvement, Reporting

## Abstract

**Background:**

There is interest internationally in improving the uptake of research evidence to inform health care quality and safety. This article focusses on guidance development from research studies as one method for improving research uptake. While we recognise that implementation strategies on the ´demand´ side for encouraging the uptake of research are important, e.g. knowledge brokers and university-practice collaborations, this article focusses on the ´production´ aspect of how guidance development is reported and the consequent influence this may have on end-users´ receptivity to evidence, in addition to other demand-side processes.

**Main text:**

The article considers the following question: how is guidance developed and what are the implications for reporting? We address this question by reviewing examples of guidance development reporting from applied health research studies, then describe how we produced guidance for a national study of evidence use in decision-making on adopting innovations. The starting point for reflecting on our experiences is a vignette of the guidance ´launch´ event at a national conference.

**Conclusions:**

Implications for reporting guidance development and supporting improvement are discussed. These include the need to (a) produce reporting standards for the production of guidance to match reporting standards for other research methods, (b) acknowledge the ´informal´ or emergent aspects of producing guidance and its role within a wider knowledge mobilization strategy, (c) consider guidance development from projects as part of a wider knowledge mobilization strategy, and (d) encourage a receptive environment for guidance development and use, including researcher training, durable funding to support impact, and closer relations between research and practice.

## Background

There is interest internationally in improving the uptake of research evidence to inform health care quality and safety [1–3]. This article examines guidance development from research studies as one method for improving research uptake. We consider this research question: how is guidance developed and what are the implications for reporting? To address this question, we focus on how the production of guidance from applied health research is reported in a small sample of studies and then reflect on our research team’s experiences of developing guidance. The brief review of examples of guidance development reporting from applied health research studies suggests that transparency concerning how guidance was produced could be improved. Moreover, the review suggests to us that there is an informal, somewhat ´mysterious´ aspect to guidance development, which we then explore by reflecting on how we produced guidance for a national study of evidence use in decision-making on adopting innovations. Implications for reporting guidance development and supporting improvement are then discussed. The discussion emerges from our reflections on experiences of guidance development which represents a partial view and is designed to stimulate debate. There will be other angles, points of view and experiences of guidance development and reporting, upon which we hope this article encourages further debate.

Decision-makers are increasingly demanding evidence from research that synthesises implications concerning effectiveness of interventions or change programmes along with actionable findings that can be tailored to their own context [4], including implementation considerations [5]. We recognise that knowledge mobilisation involves a ‘system’ of diverse structures and actors [6], necessitating activity at this level to influence the research to practice gap, and that system-level implementation strategies, e.g. knowledge brokers, university-practice collaborations, and research commissioned to address policy questions [7–9], are key to this endeavour. However, the reality is that emphasis continues to be placed upon the ´production´ aspect of knowledge translation, including the development of guidance. This piece therefore focusses on how guidance development is reported and the consequent influence this may have on end-users´ receptivity to evidence, in addition to other demand-side processes.

Building on recent debate in the field [10], we define guidance as systematically developed statements to aid decision-making on health system challenges. We treat the term ‘systematically developed’ as an empirical question in relation to how producers go about developing guidance, rather than equating this a priori with a fixed set of steps to follow (e.g. as found in institutional guidance such as the World Health Organization’s (WHO) approach to evaluating interventions based on systematic and qualitative reviews) [11]. Institutional producers of guidance, such as the WHO and the UK’s National Institute for Health and Care Excellence (NICE) have formalised approaches for developing and reporting guidance. However, no consensus exists on how guidance development should be reported that is derived from individually funded studies in the field of health services research.

The relative informality with which guidance is produced in this context may help to account for the ´image´ problem that guidance for improvement is sometimes ascribed [12]. Formalized approaches to developing guidance normally involve evaluating interventions based on systematic reviews. Less is known about the relevance of formalized approaches for developing guidance concerning (1) other phenomena identified through health services research not reducible to ‘interventions’, (2) the translation of evidence into recommendations that can inform decision-making, and (3) the sharing of recommendations in such a way that is likely to maximise their impact on practice. With regard to the ´production´ side of improving research uptake, key areas of consideration include the message, target audience, the messenger, knowledge transfer processes, and evaluation to inform future knowledge mobilisation strategy [13].

Our contention is that divergent approaches to developing guidance are emerging which are (a) based on findings from individual studies in health services research and are not therefore wedded to institutional approaches to guidance development, (b) increasingly required to respond to specific audiences´ needs [4–5] and producers may therefore tailor their approach to guidance development to meet those needs, and (c) where ´systematic´ steps for producing guidance have been codified these may or may not be followed in practice by guidance producers. In this debate article, we reflect on the variety of approaches being used to develop guidance, including the ´informal´ or bespoke aspects, and consider any implications for how ´systematic´ is defined and aspired to in relation to guidance development.

### Main text

#### Guidance development reporting

We reviewed a small sample (6) of the reporting of guidance development methods from studies supported by major funding bodies (Table 1). These were identified by searching research funders´ websites in North America and Europe (National Institute for Health Research in England, European Commission, Canadian Institute for Health Research, US National Institutes of Health) and reviewing other examples of guidance for improvement of which the research team were aware. These were selected by searching the websites for key terms including ´guidelines´, ´guidance´, and ´toolkits´ and reviewing some of the resulting reports, or associated journal papers, for examples of guidance for improvement derived from health services research studies. The aim was not to perform a comprehensive review, but to situate our own experiences of developing guidance in relation to other studies. Our interest was in guidance development processes and reporting from individually funded studies, rather than guidance that follows institutionally prescribed approaches such as WHO.

**Table 1:** Studies reporting guidance development reviewed

Authors	Funding body	Topic	Product	Guidance development methods
Waterman H, Tillen D, Dickson R, De Koning K. Action research: a systematic review and guidance for assessment. *Health Technol Assess* 2001;5 (23)	UK. National Institute for Health Research (NIHR) Health Technology Assessment (HTA) programme	Assessing action research proposals and projects	Tool for ´critical reflection´ based on 20 guidance questions and points for discussion. Draft guidance requiring ´field-testing´.	• Synthesis of findings in relation to study objectives • Literary sources, action researchers´ experiences, findings of review
Wong G, Greenhalgh T, Westhrop G, Pawson R. Development of methodological guidance, publication standards and training materials for realist and meta-narrative reviews: The RAMESES (Realist And Meta-narrative Evidence Syntheses: Evolving Standards) project. *Health Serv Deliv Res* 2014;2 (30)	UK. NIHR Health Services Research and Delivery Programme	Guidance on producing realist and meta-narrative reviews	Publication standards, quality standards, and teaching and learning resources for realist and meta-narrative reviews.	• Literature search • Delphi panels • Email discussion list • Methodological support to review teams • Workshops
Bennett MI, Mulvey MR, Campling N, Latter S, Richardson A, Bekker H, et al. Self-management toolkit and delivery strategy for end-of-life pain: the mixed-methods feasibility study. *Health Technol Assess* 2017;21 (76)	UK. NIHR HTA	Self-management of end-of-life pain	Self-management intervention including patient/carer support toolkit and nurse-led educational programme.	• Initial scoping work, inc. 6 patient/carer interviews • Evidence synthesis, inc. key learning from studies conducted by the team • Interviews / focus groups to refine and detail intervention
Carpenter D, Nieva V, Albaghal T, et al. Development of a Planning Tool to Guide Research Dissemination. In: Henriksen K, Battles JB, Marks ES, et al., editors. Advances in Patient Safety: From Research to Implementation (Volume 4: Programs, Tools, and Products). Rockville (MD): Agency for Healthcare Research and Quality (US); 2005 Feb. Available from: https://www.ncbi.nlm.nih.gov/books/NBK20603/	US. Agency for Healthcare Research Quality (AHRQ)	Research dissemination	Research dissemination planning tool	• Literature review • Review of current tools • Expert review of draft tool • Testing tool with end users (patient safety researchers)
Goering P, Ross S, Jacobson N. Developing a guide to support the knowledge translation component of the grant application process, *Evidence & Policy*, 2010;6.1: 91–102.	Canadian Health Research Foundation (CHSRF), the Canadian Institutes for Health Research (CIHR), National Institute for Health Research Service Delivery and Organization (SDO) Research and Development Programme, and the Netherlands Organization for Health Research and Development	Assessing research grant applications´ knowledge translation plans	Guide for assessing health research knowledge translation plans	• Developed draft guide based on existing literature and team’s expertise • Cognitive interviews to assess applicant and reviewer responses to the guide • Revising draft based on interviews (consensus method among team used to decide on revisions)
Janet E Anderson, Glenn Robert, Francisco Nunes, Roland Bal, Susan Burnett, Anette Karltun, Johan Sanne, Karina Aase, Siri Wiig, Naomi J Fulop, QUASER team, Translating research on quality improvement in five European countries into a reflective guide for hospital leaders: the ‘QUASER Hospital Guide’, *International Journal for Quality in Health Care*, mzz055, 10.1093/intqhc/mzz055	European Community’s Seventh Framework Programme (FP7)	Hospital leaders´ quality improvement work and strategies	Reflective guide for developing quality improvement strategy aimed at hospital leaders	• Three expert group workshops on need/approach, emerging design, and feedback on prototype • Report summarising workshop findings and implications for design

**Table 2:** Summary of the DECIDE guide

***Purpose:*** Support the use of evidence in decision-making about innovation by providers and commissioners of care. ***Format:*** The guidance is a free-to-download PDF with interactive features (users can click to open boxes with case study examples and to navigate between different sections of the guide). It is organised around six key themes, and questions for decision-makers, using the visual metaphor of the ‘long and winding road’ of decision-making on introducing innovations: • Definition - can the innovation and its potential impact be clearly described? • Evidence - what evidence is available in relation to the innovation? • Stakeholders - who will be involved in decisions and how? • Drivers - what are the key external and internal drivers for introducing innovation? • Organisation - what organisational factors should be considered during decision-making? • Implementation- can likely barriers and enablers to implementation be anticipated early in decision-making? Examples from three qualitative case studies of ‘real-world’ decision-making on innovation undertaken during the DECIDE study (stroke reconfiguration, ‘virtual’ clinics for glaucoma outpatients, and national guidance on referral for suspected cancer) were used to illustrate the themes throughout the guide. The key questions are repeated in a summary ‘checklist’ at the end of the guide, designed for users to review whether they have considered each question in their decision-making. ***Evidence:*** The final guide produced was informed by a consultative, rather than co-design, approach with potential end-users of the guidance. The steps involved in developing the guidance included: (1) scoping interviews to identify decision-makers´ needs; (2) reviewing related guidance; (3) summarising key study findings, including a published systematic scoping review, [34], and assessing their practical implications for decision-making practice; (4) stakeholder feedback on content, style, and usability of guidance iterations through interviews (nine) and group workshop (32 attendees); and (5) consulting a design agency who developed an interactive PDF design in response to our requirements. Multiple iterations of the guidance were produced in response to stakeholder feedback. ***Availability:*** The guidance is freely available to download from: https://www.ihpo.manchester.ac.uk/research/projects/decide/decide-guidance/	

In the US, a research dissemination planning tool was developed by reviewing existing literature and tools, and organising expert review of the draft tool and end user testing [14]. In Canada, a guide for assessing knowledge translation plans was developed by drafting guidance, based on existing literature and the team’s expertise, then conducting ‘cognitive interviews’ to assess end-users’ responses [15]. Revisions to the guidance were based on a ‘consensus method’ within the team and reference to a project advisory committee. In the UK, standards for reporting evidence syntheses were informed by literature searches, team’s expertise, Delphi panels, email discussion list, and workshops [16]. Another research team undertook patient/carer interviews, evidence synthesis including learning from the team’s previous studies, and interviews/focus groups to refine an intervention [17]. Guidance for assessing action research proposals was developed by synthesising study findings, including a literature review, and combining this with their views as action researchers [18]. An EU-funded study on quality improvement strategies in five countries used stakeholder workshops to inform a reflective guide for hospital leaders [19].

We now summarise briefly how guidance development is reported from these studies. These examples suggest to us the importance of the ‘informal’ aspects of developing guidance. First, ‘co-production’ of guidance appears sometimes to be used to ‘confirm’, rather than develop or change, authors’ established ideas for guidance. One study reported that, while they obtained user feedback on their knowledge translation guide through interviews, it was developed initially by one researcher, then ´revised and developed based on team review and discussion´ [15]. Second, authors’ experiences are afforded similar status to external evidence. This includes citing learning from their previous projects [17], and using their ‘own content expertise of the topic area’. [16] This suggests to us a need for guidance producers to use a wider range of knowledge than one’s own research. Third, there can be something mysterious or opaque about how guidance is informed by evidence; one report refers to data from different sources being ‘channelled and collated contemporaneously’ to develop quality standards [16]. Fourth, guidance is presented in many forms, from lists of questions or tables of quality standards at the end of a report [16], to ‘draft’ guidance that ‘require field testing’, [18] and practical resources or toolkits used in health service interventions [17].

We now reflect on our experiences of producing guidance for a national study (Table 2) to focus on the ‘informal’ processes in our own example, to unpick the mysterious aspects of developing guidance apparent in other studies´ reporting. We begin with a vignette of the guidance ‘launch’ at a conference workshop.

#### Vignette: guidance launch evokes cynicism

A month or so after submitting our final report to the research funder, we presented the DECIDE guide at a national conference workshop on translating academic findings into practical guidance. In one of the presentations, the audience were asked to consider what stakeholders’ most common view of toolkits might be from a range of options (warmth, cynicism, ambiguous, fad). The majority of the audience chose ‘cynicism’, reflecting the views identified in the research findings presented [12]. Some of the feedback we received from the table discussions reflected this cynicism about the role of guidance in health care improvement. There was the challenge of being able to reach practitioners, as they do not necessarily read email. Then, a challenge came of how to get people “on board”. There was the challenge of how to get people to act on the guidance versus merely reading it. And, even if local interest in the guidance could be secured, there was the challenge of how to spread the guidance beyond the immediate context. A further problem was raised of identifying who was responsible for implementing and disseminating guidance. Whose role was it? Scholars should not lead implementation (they didn’t have the skills necessary or the inclination). There was a need to create people in charge of implementing guidance. Who should be paying for it, research funders? It sounds expensive, too*.*

[Reflections on conference workshop, July 2018]

We suggest that the approach to reporting guidance development processes helps to account for such cynicism amongst some of the researchers and practitioners present. As some examples we reviewed showed, this includes sketchy reporting, reliance on personal experience, and variation in how guidance is presented.

### DECIDE guidance development methods

Our broad approach for producing the guidance was planned in advance and published in a study protocol [20]; this included recognition of suggested strategies for improving the use of evidence by decision-makers [13]. In practice, many of the steps involved in developing the guidance emerged during the course of the research project as we reflected on our findings and considered how best to present them to inform real-world practices of decision-making (including use of stakeholders´ views to support this endeavour). The emergent guidance development methods (Table 2) led us to include: concise, visual, practical examples; less ‘academic’ text; questions posed from decision-makers´ perspectives; and more prominent questions for decision-making addressed by including a checklist for practitioners.

Given the emergent aspect involved in developing guidance that we, and the examples reviewed above highlight, we now examine in more detail these ‘informal’ practices, which may not be captured by reporting standards. When reflecting on our own efforts to produce guidance, the insight from the sociology of science literature that “scientists and observers are routinely confronted by a seething mass of alternative interpretations” [21], very much resonated with our experience. These alternatives then need to be resolved somehow, either ´informally´ or in a way that is not pregiven by plans. While we are calling for the methods through which guidance is developed to be made more explicit by producers, we would caution against rationalizing these ‘informal’ or emergent processes of guidance development into a logic by which guidance is produced for research funders and practitioners that we might have acted in accordance with, but did not follow in practice [22]. For example, it is sometimes reported in relation to qualitative thematic analysis that differences in opinion among researchers were ‘resolved through debate’ [23] but this glosses over the quality of social interaction, including the role of power dynamics, novelty achieved through dialogue, and hesitancy about how to ‘go on’. The urge to ‘cover up the traces’ of, rather than acknowledge, the messy process by which knowledge is produced can be partly linked to the privileging of rationalism in Euro-American epistemology [24].

Such ‘informal’ or emergent processes played an important role in the development of guidance from our study, as these informed: decisions about which stakeholder comments on the guidance were within scope; balancing the space used for our findings, case study examples, and questions for decision-makers; language style and tone; and arranging the guidance around the metaphor of the ‘long and winding road’ of decision-making. We experienced hesitancy, however, in making such decisions. The hesitancy we experienced might reflect a lack of consensus about how to produce guidance. It could also be linked to the lack of a typical style or format for producing guidance, in the way that journals or research funders have a ‘house’ style that helps orient the ‘epistemic tinkering’ [25] needed to situate new insights in relation to current knowledge. That said, a lack of guidance on reporting may liberate producers to consider novel formats and language to communicate content in creative ways. We suggest that it is important to be explicit about the methods used for producing guidance; reporting standards would improve transparency concerning how guidance was produced, similar to reporting items used for other research methods [26–28]. This is not to argue for homogeneity concerning the development of guidance, as it differs from a systematic review and can take different forms depending on the context of improvement being addressed, but for transparency concerning what was involved in its production.

From the review of guidance development in this paper and our own experience, we encourage further debate about whether transparency in guidance development reporting could be improved by routinely including: (a) a statement of evidence on which guidance is based, distinguishing between use of authors’ research and others’ findings, (b) the approach used to gather stakeholder or end-user feedback on guidance need, format and content, (c) how external feedback was translated into change recommendations (e.g. consensus development), (d) any constraints that precluded use of feedback (e.g. out of scope) and how these were determined, and (e) specify where the guidance can be accessed by end-users as a standalone product.

It is also important to acknowledge the interactive, often informal, practices through which knowledge is developed that may not be captured in rationalized accounts. This fits with a ´complexity perspective´ on guidance development which acknowledges the multiple processes influencing the behavior of health care interventions and contexts, and the need for guidance to reflect these [29]. The interpretative work in developing guidance appears analogous to ‘abduction’ [30] in qualitative research whereby data to inform the product’s development (e.g. end-user feedback) are interpreted with ‘theoretical sensitivity’, that is, using knowledge and experience gained through the research study to inform how the feedback is addressed (e.g. our reading of innovation processes differed from some of the participants we gained feedback from). In our study, the sources of ‘sensitivity’ were broader than experience associated with conducting the research because they extended to the external design agency’s knowledge, which provided a steer on ‘what works’ visually and functionally, as well as technical constraints. In future guidance development, we would suggest widening the domain of ‘sensitivity’ to incorporate a range of expertise in guidance development. For example, to overcome differences in interpretation of innovation, we would run more interactive feedback sessions (referred to in Table 2) in which both researchers and end-users can share how and why they interpret key ideas discussed in the guidance as they do.

## Conclusions

Cynicism about guidance might be expected given the complexity of the health care environments that it seeks to improve. Written reports of research findings, as well as journal papers, are often received with cynicism concerning their relationship to improving practice. These academic outputs are not necessarily cheaper or more efficient ways of publishing findings, either. The article processing charge for publishing an open access article can be up to £3490 [31] and, in our experience, publishing findings can consume considerable academic time and resources, potentially reducing their timeliness. This is due partly to the need to write in accordance with journals´ or research funders´ conventions (especially making the case for the contribution to knowledge that differs by the audience you are writing for) and to navigate often lengthy peer review processes, with no guarantee of success. We were able to develop and produce guidance summarizing the study findings and their implications for practice in nine months, with the guidance freely available to download from a university webpage six weeks later. We acknowledge that guidance for improvement has an ‘image’ problem, and are calling for guidance producers to be transparent about the formal and informal processes by which guidance is made (e.g. within a brief structured statement of evidence on which guidance is based). However, we suggest that non-traditional outputs have an important role in knowledge mobilization strategies, given the challenges associated with achieving impact through traditional forms of reporting findings (e.g. journal papers, funder reports). As part of the strategy of identifying the target audience(s) for research findings [13], we suggest producers of research consider how the medium can be tailored to each audience. For example, open access journal articles may be more appropriate for academically-oriented audiences, while other forms including lay summaries and carefully crafted questions to help decision-makers with relating research to their own context, may be needed for other audiences.

To support the effective mobilization of guidance from research, a number of issues for policy and practice need to be addressed. Firstly, reporting standards for producers of guidance need to be developed that are appropriate for this form of research output. Secondly, the particular skills required by researchers (or others with this role) to develop and mobilize guidance from research need to be identified and matched to training opportunities. Thirdly, research bids that include guidance development need to acknowledge the time needed to not only disseminate guidance, but also have an impact on practice. This longer time horizon would align with the UK’s audit of research quality, ‘Research Excellence Framework’, which aims to capture impact from research over a 20 year period (2000–2020). Fourthly, however, opportunities for closer relations between research and practice are being fostered through sustained funding of university-healthcare collaborations [32], improvement fellowships, embedded research [33], and rapid service evaluation centres. We suggest the importance of acknowledging both the formal and informal processes involved in developing guidance for improvement (e.g. being explicit about the methods through which guidance is produced and also developing relationships to enable co-design of guidance with stakeholders to be able to wear decision-makers´ shoes). In accordance with a ‘systems’ approach for addressing the research-to-practice gap [10], improving collaborative leadership skills and access to durable funding to support such relationships matter as much as the medium through which practice implications from research are shared.

## Data Availability

The dataset supporting the conclusions of this article is included within the article.

## Abbreviations

DECIDE: DEcisions in health Care to Introduce or Diffuse innovations using Evidence
